# Enhancing Hemophilia A Care Through Home-Based Prophylaxis: Real-World Outcomes of a National Patient Support Program in Mexico

**DOI:** 10.3390/jcm15031217

**Published:** 2026-02-04

**Authors:** Israel Rico-Alba, Alberto Retana Guzmán, Horacio Marquez-Gonzalez, Jessie Nallely Zurita-Cruz

**Affiliations:** 1 Novo Nordisk, Mexico City 11560, Mexico; ivra@novonordisk.com (I.R.-A.); aoxr@novonordisk.com (A.R.G.); 2 Epidemiological Research Unit, Hospital Infantil de México Federico Gómez, Ministry of Health (SSA), Mexico City 06720, Mexico; horaciohimfg@gmail.com; 3 Facultad de Medicina, Universidad Nacional Autónoma de Mexico, Hospital Infantil de Mexico Federico Gómez, Mexico City 06720, Mexico

**Keywords:** hemophilia A, recombinant factor VIII, prophylaxis, home infusion, patient support program, treatment adherence, bleeding rate, real-world evidence, public–industry collaboration, Mexico

## Abstract

**Background/Objectives:** Patient Support Programs (PSPs) are increasingly used to support treatment adherence and continuity of care in chronic, high-cost conditions. In hemophilia A, consistent prophylaxis is essential to prevent bleeding episodes and long-term joint damage. In Mexico, disparities in access to treatment have encouraged the development of public–industry collaborative models. The objective of this study was to describe the structure, implementation, and operational characteristics of a PSP delivering home-based prophylactic treatment for individuals with hemophilia A in Mexico, and to compare annual bleeding rates according to factor VIII dosing adequacy. **Methods:** A cross-sectional, retrospective analysis was conducted using fully anonymized operational data from the PSP registry between January 2023 and March 2024. Variables included infusion location and administrator, prescribed and used doses, weekly infusion frequency, program incorporation and discontinuation, geographic coverage, and bleeding events. Annual bleeding rates were compared across dosing categories using Poisson regression models with patient-years as an offset. **Results:** A total of 1173 patients contributed 16,331 infusion records. Participants were predominantly male (99.8%), with a median age of 26 years; 71.8% had severe hemophilia. Home infusion accounted for 92.0% of administrations, primarily self-administered or caregiver-delivered. The median prescribed and used monthly doses were 18,000 IU and 16,000 IU, respectively, with dose concordance observed in 66.8% of records. Only 40.7% of patients achieved the recommended prophylactic frequency of three infusions per week. Geographic coverage increased from 62.5% to 71.9% of states. The overall annualized bleeding rate was 2.24 bleeds per patient-year. When stratified by dosing adequacy, patients receiving doses consistent with clinical recommendations showed the lowest bleeding rate (0.18 bleeds per patient-year), compared with those with overdosing (3.84) and underdosing (6.68), with statistically significant differences between groups. Knees, elbows, and ankles were the most frequently affected sites. **Conclusions:** This PSP achieved broad national reach and high adoption of home-based infusion. The observed dose-dependent differences in bleeding rates underscore the clinical relevance of appropriate prophylactic dosing within structured support programs and support the value of PSPs in strengthening treatment continuity in middle-income settings.

## 1. Introduction

Hemophilia A is an X-linked hereditary coagulation disorder caused by factor VIII deficiency and is classified as a rare disease, with an estimated prevalence of approximately 1 in 5000 male births and substantial clinical heterogeneity. Individuals with severe hemophilia are at high risk of spontaneous bleeding, progressive musculoskeletal damage, and long-term disability if not adequately treated [1,2]. International clinical guidelines consistently recommend prophylactic factor replacement as the standard of care, given its well-established role in reducing bleeding frequency, preventing hemophilic arthropathy, and improving long-term quality of life [3,4,5]. Economic evaluations conducted in both high- and middle-income settings have shown that prophylaxis is more cost-effective than on-demand treatment, particularly when initiated early in life [6].

Over the past several decades, major advances in hemophilia care have included the transition from plasma-derived products to recombinant factor concentrates, the development of extended half-life formulations, and the introduction of non-factor therapies. However, in Mexico and other Latin American countries, access to these advances remains uneven. Geographic disparities, limited availability of specialized care, variability in public-sector procurement processes, and differences in coverage policies continue to influence treatment delivery. Although therapeutic availability for hemophilia has progressively improved in Mexico over the last three decades, persistent challenges remain related to territorial equity, standardization of care, and the absence of comprehensive national registries [1,2].

Half a century ago, home-based prophylactic treatment was recommended as a strategy to improve outcomes in individuals with hemophilia, particularly in countries with universal health coverage and robust health-system infrastructure. In contrast, middle-income countries have historically relied on facility-based care, and home care programs have been largely limited to highly prevalent chronic conditions. In recent years, public–private collaborative models have emerged as pragmatic approaches to strengthen treatment continuity and reduce structural barriers to care. In this context, a patient support initiative was implemented in Mexico in collaboration with public healthcare institutions to facilitate home delivery of recombinant factor VIII for individuals receiving prophylactic treatment, supported by monitoring and educational components.

Patient Support Programs (PSPs) developed by the pharmaceutical industry have been increasingly incorporated into the management of chronic and rare diseases, with the aim of improving adherence, supporting self-management, and reducing treatment interruptions [7]. Evidence suggests that well-structured public–private partnerships can enhance health-system capacity, improve access to care, and positively influence patient-reported outcomes [8]. Nevertheless, despite their growing implementation, PSPs are infrequently subjected to formal evaluation, and only a minority have been systematically assessed in peer-reviewed literature, particularly in middle-income settings.

Existing publications from Latin America have largely focused on pharmacologic coverage, cost-effectiveness analyses, and clinical practice guidelines for hemophilia, with limited attention to the operational characteristics and real-world performance of PSPs embedded within public health systems [9,10,11]. As a result, there is a paucity of evidence describing how such programs function in routine practice and how they may influence treatment delivery and clinical outcomes.

Therefore, the objective of this study was to describe the structure, implementation, and key components of a patient support program for individuals with hemophilia A operating in collaboration with public healthcare institutions in Mexico, and to compare annual bleeding rates according to factor VIII dosing adequacy among patients enrolled in the program, while all other program characteristics and outcomes were analyzed descriptively.

## 2. Materials and Methods

### 2.1. Study Design

This was a cross-sectional, retrospective study designed to describe the implementation, operational characteristics, and clinical outcomes of a Patient Support Program (PSP) for individuals with hemophilia A in Mexico. The analysis was based on fully anonymized secondary operational data obtained from the PSP registry and covered the period from January 2023 to March 2024.

The PSP was initiated in 2020 by Novo Nordisk in collaboration with public healthcare institutions in Mexico. The program was developed to support individuals with hemophilia A receiving prophylactic treatment by facilitating home delivery of recombinant factor VIII, combined with monitoring and educational support. Operational data were generated as part of routine program activities and subsequently used for descriptive and analytical purposes in this study.

### 2.2. Data Sources

The data used in this study were obtained from the internal digital registry of the Patient Support Program (PSP). This registry is routinely maintained as part of program operations and is used to document medication delivery, infusion practices, and patient follow-up activities. Data are entered prospectively by patients, caregivers, or program personnel using standardized reporting formats.

The registry includes detailed information on infusion records (date, location, and administrator), prescribed and used doses of recombinant factor VIII, prophylactic in-fusion frequency, reported bleeding events, and program participation indicators such as patient incorporation, discontinuation, and geographic distribution. All data were extracted in anonymized form prior to analysis, and no identifiers allowing patient reidentification were available to the investigators.

For the present analysis, data corresponding to the period from January 2023 to March 2024 were retrieved. Data extraction was limited to variables relevant to the study objectives and was performed solely for research purposes, without altering routine program operations.

### 2.3. Study Population

The analysis included all patients enrolled in the PSP during the study period who had available infusion records, dosage information, and delivery logs. Eligible participants were individuals with a confirmed diagnosis of hemophilia A who were enrolled in the program for prophylactic treatment and had at least one documented infusion record during the observation period. Patients were excluded if they lacked infusion records for the entire study period or if treatment logs were incomplete or invalid.

### 2.4. Variables and Definitions

Infusion-related variables described the location and administrator of recombinant factor VIII. Home infusion was defined as any infusion performed at the patient’s residence. Hospital administration referred to infusions carried out within healthcare facilities. For a small proportion of records, the location of administration could not be determined. The individual administering the infusion was classified as the patient, an informal caregiver, or healthcare personnel.

Dosage-related variables included the prescribed dose and the used dose of factor VIII. The prescribed dose corresponded to the number of international units (IU) recommended by the treating physician, while the used dose reflected the IU recorded by patients in their infusion logs. Dose concordance was defined as agreement between prescribed and used doses, whereas underdosing and overdosing were defined as used doses below or above the prescribed amount, respectively.

Adherence to prophylactic treatment was assessed using two complementary dimensions. Dose concordance was evaluated as described above, and adherence to infusion frequency was assessed based on compliance with the recommended schedule of three prophylactic infusions per week, according to national clinical guidelines. Patients meeting this frequency criterion were classified as having adequate prophylactic frequency adherence.

Program coverage variables included the number of active patients, defined as individuals with at least one documented infusion during the study period, as well as patient incorporation and discontinuation during follow-up. Geographic coverage was defined as the number and proportion of states with at least one active participant enrolled in the program.

Bleeding events were defined as any reported hemorrhagic episode requiring treatment with factor VIII. The annual bleeding rate (ABR) was calculated as the total number of bleeding events divided by patient-years of follow-up.

### 2.5. Data Processing and Quality Control

Data underwent a structured cleaning and validation process to ensure accuracy and completeness. Duplicate infusion entries were identified and removed. All records were examined to detect missing or inconsistent information, such as absent details regarding the site of administration. Dosage values were also reviewed to confirm that they fell within physiologically and clinically plausible ranges; for example, entries reporting 0 IU for a prophylactic infusion were flagged for verification. Outliers were assessed for plausibility and were retained unless clear evidence indicated that the values were erroneous.

### 2.6. Statistical Analysis

Descriptive statistics were used to summarize patient characteristics, infusion practices, and program coverage. Continuous variables are presented as medians with interquartile ranges, while categorical variables are presented as frequencies and proportions.

To address reviewer concerns regarding the interpretation of bleeding outcomes, annual bleeding rates were compared across dosing categories (adequate dosing, underdosing, and overdosing) using Poisson regression models with log-transformed patient-years as an offset, allowing estimation of incidence rate ratios. Statistical significance was defined as a two-sided *p*-value < 0.05. All analyses were conducted using STATA version 16 (StataCorp, College Station, TX, USA).

### 2.7. Ethical Considerations

This study was conducted in accordance with the ethical principles of the Declaration of Helsinki. The analysis was based exclusively on fully anonymized secondary operational data obtained from the PSP registry, originally collected for routine program monitoring and logistical purposes. No identifiable personal information was accessed, and no direct contact or interaction with participants occurred.

According to Mexican national regulations governing health research, specifically the Regulation of the General Health Law on Health Research, retrospective analyses of anonymized secondary data that do not involve interventions or pose risk to participants are classified as research without risk and are exempt from review and approval by a Research Ethics Committee. Therefore, ethical approval was not required for this study.

## 3. Results

### 3.1. Patient Characteristics

During the study period, a total of 1173 patients were included, contributing 16,331 infusion records. Most participants were male (99.8%), with a median age of 26 years. The most frequent age group was 15–44 years, accounting for 60.7% of the cohort. Severe hemophilia A was the predominant phenotype, observed in 71.8% of patients (Table 1).

Regarding health coverage, 70.3% of patients reported employment-based insurance, including coverage for spouses and children. Pension-based coverage was reported by 14.8%, school-based insurance by 7.5%, and independently paid insurance by 5.6%. Other coverage types accounted for 1.3% of cases, and information was unavailable for 0.7% of patients (Table 1).

### 3.2. Program Growth and Geographic Coverage

At the beginning of the study period, 913 patients were enrolled in the program. During follow-up, an additional 260 patients were incorporated, representing a 28.5% increase in program participation. Over the same period, 76 patients discontinued the program due to administrative or eligibility-related reasons.

Geographic coverage expanded from 62.5% (20 of 32 states) at baseline to 71.9% (23 of 32 states) by the end of the study period. Among states with active participation, 19 (82.6%) experienced an increase in the number of patients served, while a decrease was observed in three states (13.0%).

### 3.3. Infusion Practices and Treatment Administration

With respect to the location of medication administration, 92.0% of infusions were performed at home, while 6.25% were administered in hospital settings. For the remaining 1.75% of infusions (*n* = 291), the location of administration could not be determined.

Regarding the individual administering treatment, most infusions were self-administered by patients (62.3%), followed by administration by an informal caregiver (27.5%). A smaller proportion of infusions were administered by healthcare personnel. Prophylactic treatment accounted for 97.1% of all recorded infusions, reflecting the predominance of prophylaxis-based management within the program (Table 2).

### 3.4. Dosage Patterns and Adherence

The median prescribed monthly dose of recombinant factor VIII was 18,000 IU, while the median used dose was 16,000 IU. Dose concordance between prescribed and used doses was observed in 66.8% of infusion records.

When adherence was assessed using infusion frequency, only 40.7% of patients met the recommended schedule of three prophylactic infusions per week (Table 2). This finding indicates that adherence based on dosing accuracy and adherence based on infusion frequency captured distinct aspects of real-world prophylactic behavior.

### 3.5. Bleeding Events and Annual Bleeding Rate

A total of 3283 bleeding episodes were reported during the study period. The overall annualized bleeding rate (ABR) was 2.24 bleeds per patient-year.

**Figure 1:**
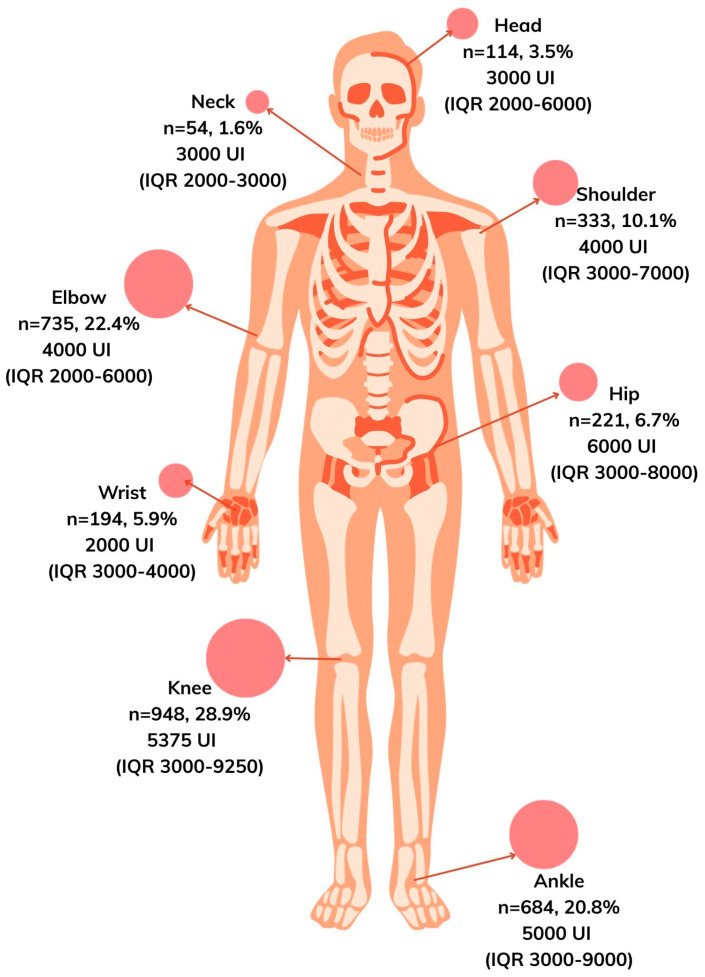
Anatomical distribution and treatment dose of reported bleeding events among patients with hemophilia A.

The knee was the most frequently affected anatomical site (28.9%, *n* = 948), followed by the elbow (22.4%, *n* = 735) and the ankle (20.8%, *n* = 684). Less frequent bleeding sites included the shoulder, hip, wrist, head, and neck (Figure 1).

When annual bleeding rates were stratified according to dosing adequacy, marked differences were observed. Patients receiving doses consistent with clinical recommendations exhibited an ABR of 0.18 bleeds per patient-year, compared with 3.84 bleeds per patient-year among patients with overdosing and 6.68 bleeds per patient-year among those with underdosing. Inferential analysis using Poisson regression demonstrated statistically significant differences in bleeding rates across dosing categories.

## 4. Discussion

### 4.1. Principal Findings

This study provides a real-world description of a national Patient Support Program (PSP) delivering recombinant factor VIII to individuals with hemophilia A in Mexico. Over 15 months, the program enrolled 1173 patients and documented 16,331 infusions, with growth in participation and expansion of geographic coverage. Most participants were young adult males with severe hemophilia, and 92.0% of infusions were administered at home, predominantly by patients or informal caregivers. Prophylaxis accounted for 97.1% of infusions, although only 40.7% adhered to the recommended three-times-per-week regimen, and dose concordance was observed in 66.8% of records. Clinically, the overall annualized bleeding rate was 2.24 bleeds per patient-year. When bleeding outcomes were stratified according to dosing adequacy, marked differences emerged: patients receiving doses consistent with clinical recommendations exhibited the lowest annual bleeding rate (0.18 bleeds per patient-year), whereas those with overdosing and underdosing experienced substantially higher rates (3.84 and 6.68 bleeds per patient-year, respectively).

The demographic and phenotypic characteristics observed in this study are consistent with global reports describing populations living with hemophilia A. Severe hemophilia often represents approximately 60–80% of treated cohorts, similar to the 71.8% reported here [8,12]. Joint bleeding distribution—primarily knees, elbows, and ankles—also reflects classical patterns documented in observational studies and registries [13].

Our overall ABR of 2.24 aligns with international prophylaxis outcomes using standard half-life factor VIII. Real-world cohorts from Europe and North America report median ABRs between 2 and 4 in adults receiving conventional prophylaxis, with lower bleeding rates typically reported in high-intensity or PK-guided individualized regimens [14,15]. Pediatric cohorts starting prophylaxis early have shown even lower ABRs (≤1.3), although these outcomes generally require structured multidisciplinary follow-up not yet universally available in Latin America [16]. The very low ABR observed among patients with adequate dosing in our cohort approaches values reported in intensively managed populations, whereas the substantially higher ABRs among patients with underdosing or overdosing underscore the clinical relevance of appropriate prophylactic dosing beyond total factor consumption.

Regional studies also underscore ongoing disparities in access and clinical outcomes. A systematic review from Brazil identified wide variability in ABR among patients with severe hemophilia A and emphasized inconsistent dosing and suboptimal adherence as key barriers [17]. Similarly, analyses from Latin American countries highlight gaps in supply continuity, regional inequality, and limited integration of home-based infusion programs [18]. Our stratified findings extend these observations by showing that deviations from recommended dosing—particularly underdosing—were associated with a substantially higher bleeding burden, even within the context of a structured PSP.

### 4.2. Program Performance and Adherence Dimensions

Home-based infusion and self-management are core components of contemporary hemophilia care and have been associated with reduced hospitalization and improved participation in school and work activities [19]. Structured patient support programs—particularly those combining home delivery, training, and psychosocial support—have been described as strategies that can strengthen adherence and reduce bleeding risk [20,21]. The high proportion of home infusions (92.0%) observed in this study is consistent with European home-care delivery networks, including Italian experience showing improved treatment continuity and reduced logistical burden through coordinated home delivery and monitoring [22].

Adherence to prophylactic regimens is multifactorial and cannot be fully characterized by dose concordance alone. In our cohort, dose concordance between prescribed and used doses was observed in 66.8% of infusion records; however, when adherence was assessed using infusion frequency, only 40.7% of patients achieved the recommended schedule of three prophylactic infusions per week [23]. These findings highlight that dose concordance and infusion frequency represent complementary dimensions of adherence, and that infusion regularity is a clinically important determinant of prophylactic effectiveness.

### 4.3. Strengths and Limitations

This study has several limitations. First, the cross-sectional, retrospective design limits causal interpretation. Second, infusion and bleeding data were self-reported, introducing potential reporting bias. Third, the study lacked clinical endpoints such as joint scores or quality-of-life measures, which would strengthen the evaluation of long-term benefit. Fourth, the absence of a comparison group limits conclusions regarding relative effectiveness. Additionally, the lack of data on factor VIII trough levels and the absence of pharmacokinetic-guided dosing information limit insight into individualized dosing decisions and their relationship with bleeding outcomes. Finally, findings may not generalize to patients not engaged in structured programs.

## 5. Conclusions

This study describes the real-world implementation of a national Patient Support Program delivering recombinant factor VIII to individuals with hemophilia A in Mexico through collaboration with public healthcare institutions. The program achieved broad geographic reach and high adoption of home-based prophylactic infusion within a middle-income health system.

Importantly, annual bleeding rates differed substantially according to factor VIII dosing adequacy, with lower rates observed among patients receiving doses consistent with clinical recommendations. These findings underscore the relevance of appropriate dosing and infusion regularity as key components of effective prophylactic care.

Overall, this program illustrates how structured Patient Support Programs may contribute to treatment continuity and patient autonomy in resource-constrained settings, while highlighting the need for ongoing efforts to optimize adherence and dosing strategies.

## Figures and Tables

**Table 1:** Description of the general characteristics of the patients included in the study.

	Variables	All *n* = 1173
Age, years		
	Median (IQR)	26 (16, 36)
Age group, *n* (%)		
	<1 year	50 (4.3)
	1 to 4 years	29 (2.5)
	5 to 14 years	233 (19.9)
	15 to 44 years	712 (60.7)
	45 to 64 years	133 (11.4)
	>65 years	16 (1.3)
Sex, *n* (%)		
	Female	2 (0.2)
	Male	1171 (99.8)
Phenotype Classification, *n* (%)		
	Mild (5–40% factor activity)	59 (5.0)
	Moderate (1–5% factor activity)	271 (23.1)
	Severe (<1% activity)	841 (71.8)
Medication administrator, *n* (%)		
	Healthcare personnel	94 (8.1)
	Informal primary caregiver	322 (27.5)
	Patient	731 (62.3)
	Unknown	26 (2.2)
Health insurance		
	Employment	70.3%
	Pensioners	14.8%
	Students	7.5%
	Individually purchased	5.6%
	Other types	1.3%
	Unknown	0.7%

**Table 2:** Description of the treatment administered to the patients during the study period.

	Variables			All *n* = 16,331
Type of administration				
	Prophylaxis, *n* (%)			15,841 (97.1)
		Monthly dose, IU; Median (IQR)		180,000 (12,000 to 24,000)
	On-demand, *n* (%)			490 (2.9)
		Monthly dose, IU; Median (IQR)		18,000 (7750 to 30,000)
Weekly infusions, *n* (%)				
	Median (IQR)			2.25 (2 to 3)
		Intervals, *n* (%)		
			0–0.75	522 (3.2)
			1.0–1.75	1959 (12.0)
			2.0–2.75	5317 (32.6)
			3.0–3.75 (medical recomendation)	6644 (40.7)
			4.0–6.0	415 (2.5)
			Unknown	1474 (9.0)
Adherence to medical indications				
	Adequate			10,909 (66.8)
	Underdosing			4883 (29.9)
	Overdosing			539 (3.3)

## Data Availability

The data presented in this study are available upon request from the corresponding author due to privacy concerns.

## Abbreviations

ABR	Annual Bleeding Rate
CDC	Centers for Disease Control and Prevention
IU	International Units
IQR	Interquartile Range
PSP	Patient Support Program
QoL	Quality of Life
WFH	World Federation of Hemophilia
